# High On-Treatment Platelet Reactivity in Danish Hyper-Acute Ischaemic Stroke Patients

**DOI:** 10.3389/fneur.2018.00712

**Published:** 2018-08-28

**Authors:** Charlotte L. Rath, Niklas Rye Jørgensen, Troels Wienecke

**Affiliations:** ^1^Department of Neurology, Neurovascular Centre, Zealand University Hospital, Roskilde, Denmark; ^2^Department of Clinical Biochemistry, Rigshospitalet, University of Copenhagen, Copenhagen, Denmark; ^3^OPEN, Odense Patient data Explorative Network, Odense University Hospital/Institute of Clinical Research, University of Southern Denmark, Odense, Denmark; ^4^Department of Clinical Medicine, University of Copenhagen, Copenhagen, Denmark

**Keywords:** anti-platelet therapy, cerebrovascular disease, clopidogrel, high on-treatment platelet reactivity, prevention, recurrent stroke, stroke

## Abstract

**Objective:** Early anti-platelet therapy is a cornerstone in the prevention of recurrent ischaemic stroke (IS) and transient ischaemic attacks (TIAs), although the responsiveness to anti-platelet medications varies among patients. Several studies have reported that patients with ischaemic stroke who exhibit high on-treatment platelet reactivity (HTPR) 5–10 days after antiplatelet medication onset, have an increased risk of vascular events. In this study we aim to determine the prevalence of HTPR in the hyper-acute stroke phase less than 48 h from symptom onset, after the administration of a 300 mg bolus of oral clopidogrel in a real-world setting in Danish IS and TIA patients.

**Material and Methods:** In total, 219 Danish patients with acute IS or TIA received 300 mg of oral clopidogrel on admission. Blood samples from all patients were analyzed using the VerifyNow P2Y12 system at 8–24 h after clopidogrel intake. Concomitant therapy and the intervals between ictus and blood collection, clopidogrel intake and blood collection, and blood sampling and analysis were recorded for all patients.

**Results:** HTPR in the hyper-acute stroke phase was observed in 28.8% (63/219) samples. After adjustment for age, sex, co-morbidities, and co-medications, none of the tested variables exhibited an association with HTPR or the platelet reaction unit value measured using the VerifyNow P2Y12 system.

**Conclusions:** The recognition of HTPR to specific anti-platelet agents in the hyper-acute phase after stroke may be the first step toward interventions that may further minimize the early recurrent stroke risk. Further large randomized trials including clinical outcome assessments are necessary.

## Introduction

Anti-platelet therapy is a cornerstone in the prevention of secondary ischaemic stroke (IS), and clopidogrel has been reported to lower the long-term risk of recurrent stroke with the same efficiency as acetylsalicylic acid combined with dipyridamole (1). Patients with a transient ischaemic attack (TIA) exhibit an increased stroke risk in the acute phase (2) although this risk is lowered after treatment with anti-platelet agents (3). The importance of very early treatment in both acute IS and TIA has been further established in a recent time-course analysis of existing randomized trials (4). However, the platelet function was not explored in these studies. Although the prevalence of patients with IS who exhibit high on treatment platelet reactivity (HTPR), also known as non-responders, has been evaluated in several other studies, the results have been highly variable (8–61%) (5).

Clopidogrel is an anti-platelet aggregation drug that inhibits adenosine 5-diphosphate from binding to its P2Y12 receptor on the surface of the platelet, resulting in the inhibition of platelet reactivity. This pro-drug requires biotransformation (two-step oxidation) that is catalyzed by several cytochrome P450 (CYP) enzymes. A previous study recommended that platelet reactivity should be tested at 8 h after the administration of a loading dose of 300 mg (6).

In a recent meta-analysis, it was found that the recurrent stroke or TIA risk was significantly higher in patients with HTPR (RR = 1.81), and that the HTPR prevalence after clopidogrel administration was 27% (7). However, the studies included in this meta-analysis differed with regard to the ethnicity of patients, treatment regimen, HTPR assessment technique, and treatment period at the time of HTPR testing. And importantly, the studies evaluated HTPR >3 days after treatment start.

The aim of the present study was to establish the prevalence of HTPR at 8–24 h after the intake of clopidogrel 300 mg in the acute phase (<48 h after symptom onset) after IS or TIA in a real-world setting.

## Materials and methods

This prospective cross-sectional study included patients with suspected acute IS or TIA who were admitted in our stroke unit, which is a primary treatment center receiving patients not previously seen by a physician. The study was approved by the local ethics committee of Region Zealand, Denmark (protocol number: SJ-390) and registered in clinicaltrials.gov (NCT02607358).

### Patient selection

Inclusion criteria

- Clinically suspected acute IS or TIA (acute onset of focal neurological symptoms originating from one vascular area of the brain and no other explanation of symptoms on brain CT) within the last 48 h
- Treatment with clopidogrel 300 mg on admission
- Provision of verbal and written informed consent prior to inclusion.

Exclusion criteria

- Previous clopidogrel treatment
- Hemorrhage or pathologies other than IS on brain CT
- Cancer
- Increased bleeding risk (e.g., recent major surgery) or ongoing bleeding (e.g., low hemoglobin, gastric ulcer)
- Treatment with vitamin K antagonists, novel oral anti-coagulants, and other adenosine diphosphate (ADP) inhibitors or non-steroidal anti-inflammatory drugs (NSAIDs) other than acetylsalicylic acid (ASA)
- Unmet criteria for a diagnosis of ischaemic stroke or TIA at discharge
- Abnormal haematocrit (Hct) values (range, 30–52%) or platelet counts (range, 119–502 × 10^3^/μL) as per VerifyNow P2Y12 system (Accumetrics, San Diego, California)instruction manual

### Clinical procedures

Routine blood tests were performed at the time of admission. Data pertaining to cardiovascular risk factors and demographic characteristics, including age, sex, ethnicity, the presence of ischaemic heart disease (IHD), the presence of diabetes mellitus, and the smoking status (cigarettes or pipe in any amount until 1 week before admission), were recorded for all patients. Information concerning treatment with anti-hypertensive medications and statins as well as concomitant medical therapy at the time of admission with anti-depressives [selective serotonin re-uptake inhibitors (SSRIs)] and proton pump inhibitors (PPIs) was also collected (Table 1).

**Table 1:** Baseline characteristics of patients with acute ischaemic stroke or a transient ischaemic attack.

	**All patients (*n* = 219)**	**Non-HTPR group (*n* = 156)**	**HTPR group (*n* = 63)**
Age, years (SD)	68.7 ± 12.5	67.1 ± 12.8	72.8 ± 10.9
Female sex (%)	90 (41.1)	61 (39.1)	29 (46.0)
IHD (%)	23 (10.6)	14 (9.2)	9 (14.3)
Previous stroke (%)	25 (11.5)	15 (9.7)	10 (15.9)
Diabetes mellitus (%)	22 (10.0)	11 (7.1)	11 (17.5)
Smoking (%)	55 (25.2)	45 (29.0)	10 (15.9)
Carotid stenosis (%)	9 (4.1)	6 (4.7)	3 (5.5)
Platelet count (SD)	244 ± 62	251 ± 66	227 ± 56
Hgb (SD)	9.0 ± 0.9	9.2 ± 0.8	8.5 ± 0.8
Hct (SD)	38 ± 3.9	39.1 ± 3.8	36.8 ± 3.6
CT-infarction (%)	74 (33.8)	48 (31.0)	26 (41.3)
MRI performed (%)	35 (16.0)	–	–
MRI-infarction (%)	26 (11.9)	20 (12.8)	6 (9.5)
Holter monitoring performed (%)	137 (63.1)	101 (65.2)	36 (58.1)
Atrial fibrillation (%)	34 (15.6)	23 (22.8)	11 (30.6)
Readmission (%)	23 (10.5)	16 (10.3)	7 (11.1)
Ischaemic stroke vs. TIA (%)	153 (69.9)	112 (71.8)	41 (65.1)
Anti-hypertensive treatment (%)	91 (41.7)	59 (38.1)	32 (50.8)
Statin treatment (%)	35 (16.1)	19 (12.3)	16 (25.4)
PPI treatment (%)	29 (13.2)	15 (9.6)	14 (22.2)
Anti-depressive treatment (%)	10 (4.6)	8 (5.1)	2 (3.2)

*HTPR, high on-treatment platelet reactivity; IHD, ischaemic heart disease; Hgb, hemoglobin; Hct, haematocrit; CT-infarction, sign of early ischaemic lesions on brain-CT; MRI-infarction, sign of ischaemic lesions in clinical relevant area on MRI; TIA, transient ischaemic attack; PPI, proton pump inhibitor; CT, computed tomography; MRI, magnetic resonance imaging; SD, standard deviation; HTPR group, high on-treatment platelet reactivity after the intake of clopidogrel 300 mg in the acute phase of ischaemic stroke or TIA*.

On exclusion of hemorrhage via brain imaging, all patients received oral clopidogrel 300 mg and ASA 300 mg under the supervision of a physician or nurse in order to ensure medication adherence.

An additional work-up for stroke was conducted according to local guidelines. This included carotid Doppler ultrasound; electrocardiography (ECG); 24 h heart rhythm monitoring during admission, with additional 7 day Holter monitoring in selected patients; and tests for hypercoagulable states (e.g., factor V Leiden, elevated homocysteine, fibrinolytic, and dysfibrinogenic disorders) conducted for selected patients at the discretion of the treating physician.

### Blood sampling

As per instructions of the manufacturer of VerifyNow, blood samples for the assessment of HTPR were collected in buffered sodium citrate tubes provided by the manufacturer at 8–24 h after clopidogrel Intake, primarily in the morning after an overnight fast and a rest period of at least 20–30 min. Careful antecubital venipuncture was performed by an experienced nurse or physician using a Vacutainer Safety-Lok system (Becton Dickinson, Franklin Lakes, New Jersey, USA) with a pre-attached holder and a 21-gauge syringe.

### Platelet function test

The VerifyNow P2Y12 system is a rapid platelet-function cartridge-based assay designed for direct measurement of the effects of drugs on the P2Y12 receptor. Platelet aggregation samples were analyzed 10–240 min after collection using the VerifyNow P2Y12 Assay according to the manufacturer's instructions. The results were expressed as platelet reaction units (PRU).Patients with HTPR were defined as drug-compliant patients with an *ex vivo* PRU value of >208 (8).

The VerifyNow system can evaluate clopidogrel effect in patients receiving dual antiplatelet therapy with ASA (9).

### Statistical analysis

All statistical analyses were performed using IBM SPSS software for Windows release 24 (Chicago, Illinois, USA), with assistance from the local statistical department at Zealand University Hospital. All data, except the intervals between ictus and blood sample collection and blood sample collection and analysis, were normally distributed. Differences in continuous variables between groups were analyzed using Student's *t*-test with Levine's test for equality of variances or the Mann–Whitney U test, as appropriate. For dichotomous data, the chi-square test was used, with the use of Fisher's exact test when the expected values in 2 × 2 tables were below 5.

Univariate and multivariate linear regression analyses were performed to determine independent predictors of the PRU value and HTPR. A *p*-value of <0.05 was considered statistically significant.

## Results

**Figure 1:**
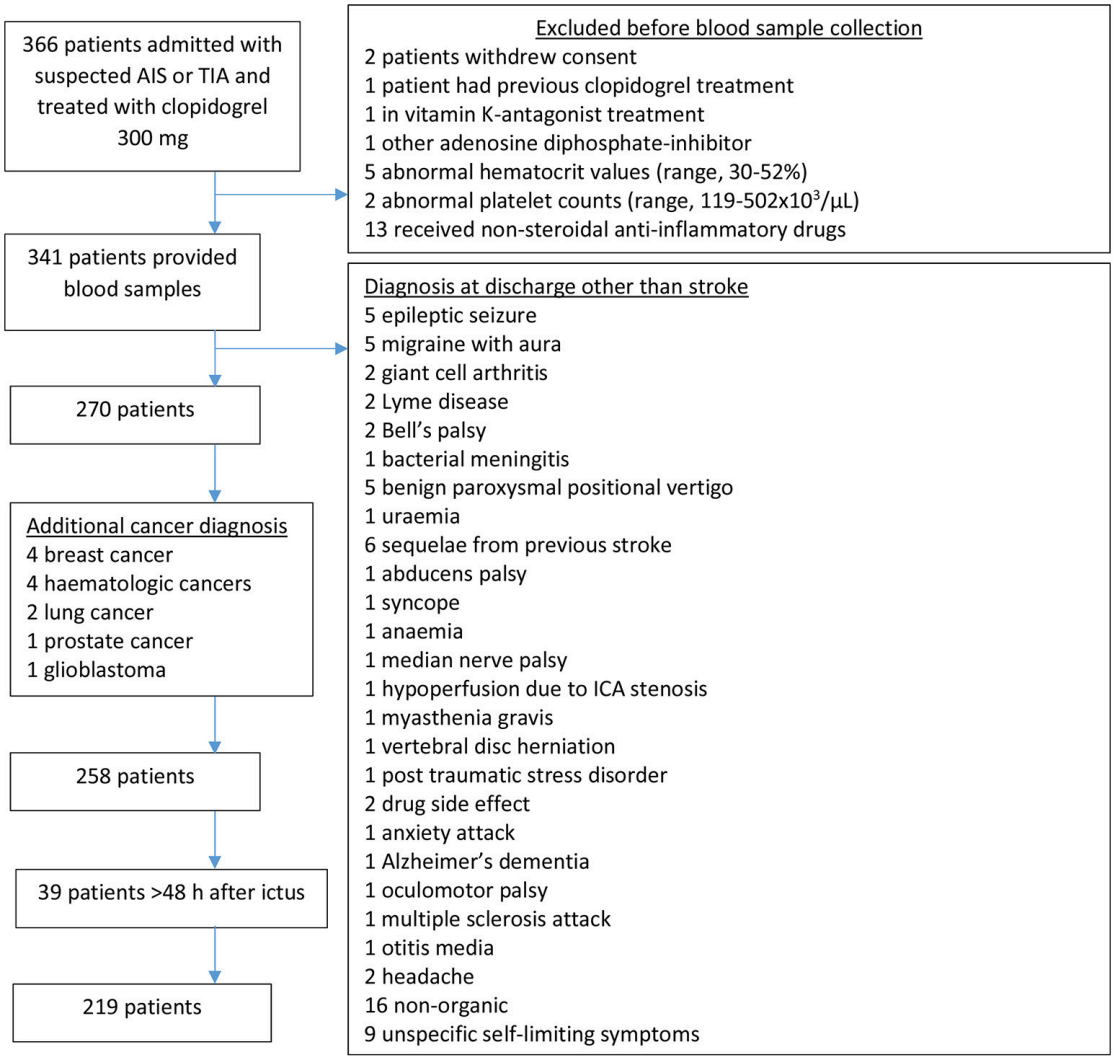
Study flow chart. The stroke unit is a primary treatment center receiving patients not previous seen by a physician. AIS, acute ischaemic stroke; TIA, transient ischaemic attack; ICA, internal carotid artery.

Of 366 patients with suspected acute IS or TIA, 219 were eligible (Figure 1).

### Factors associated with HTPR

In total, 28.8% (63 patients) exhibited HTPR (Table 1). Table 2 depicts the findings of the univariate and multivariate logistic regression analysis to determine risk factors for HTPR.

**Table 2:** Findings of univariate and multivariate regression analyses of suspected risk factors for high on-treatment platelet reactivity after the administration of clopidogrel 300 mg in the acute phase of ischaemic stroke or a transient ischaemic attack.

	**Univariate analysis**		**Multivariate analysis**	
**Risk factor**	**OR (95% CI)**	***p-*value**	**OR (95% CI)**	***p-*value**
Age	1.04 (1.01–1.07)	<**0.01**	1.03 (0.995–1.06)	0.10
Female sex	1.33 (0.74–2.40)	0.35	1.11 (0.54–2.27)	0.78
Diabetes mellitus	2.79 (1.14–6.82)	**0.03**	1.74 (0.59–5.11)	0.31
PPI treatment	2.50 (1.14–5.50)	**0.02**	2.10 (0.87–5.08)	0.10
Statin treatment	2.42 (1.15–5.09)	**0.02**	1.72 (0.69–4.33)	0.25
Anti-hypertensive treatment	1.68 (0.93–3.03)	0.09	1.17 (0.59–2.34)	0.66
IHD	1.66 (0.68–4.05)	0.27	Not tested	
Anti-depressive treatment	0.61 (0.13–2.94)	0.54	Not tested	
Smoking	0.46 (0.22–0.99)	**0.04**	0.87 (0.37–2.07)	0.76
Platelet count	0.99 (0.988–0.998)	**0.01**	0.99 (0.986–0.997)	**0.01**
Hct	0.84 (0.77–0.92)	<**0.01**	0.87 (0.79–0.96)	**0.01**

*OR, odds ratio; CI, confidence interval; PPI, proton pump inhibitor; IHD, ischaemic heart disease; Hct, haematocrit. Significant p-values (<0.05) are highlighted in bold*.

The univariate analysis revealed that increasing age, diabetes mellitus, PPI-treatment, and statin treatment were positively associated with HTPR whereas smoking, higher platelet count and higher Hct-value were negatively associated with HTPR.

In the multivariate analysis, factors from the univariate analysis with a *p*-value < 0.1 were included and adjusted for sex. In the multivariate analysis, only platelet count and Hct had a significant effect on HTPR (Table 2).

### Factors associated with the PRU value

In Table 3 is listed the different variables tested for effects on the PRU. PRU values were lower in smokers and patients without diabetes. The remaining tested binary variables did not show significant effect on the PRU.

**Table 3:** Independent samples *t*-test (top) or linear regression (bottom) on effects of different variables on the platelet reaction unit value measured using the whole-blood VerifyNow P2Y12 assay.

	**PRU mean ± SD**	**Mean difference (95% CI)**	***p*-value**
Sex			
Male	161 ± 71		
Female	164 ± 75	−3.48 (−23.2; 16.3)	0.73
Current smoking			
Smokers	143 ± 67		
Non-smokers	169 ± 74	25.0 (3.8; 48.2)	**0.02**
Diabetes mellitus			
Diabetic	198 ± 65		
Not diabetic	158 ± 73	−39.8 (−71.7; −7.9)	**0.02**
PPI-treatment			
Yes	177.1 ± 87		
No	159 ± 70	−15.6 (−51.8; 17.1)	0.31
Anti-hypertensive treatment			
Yes	165 ± 84		
No	160.1 ± 64	−4.4 (−25.1; 16.3)	0.67
Statin treatment			
Yes	173 ± 93		
No	160 ± 69	−13.4 (−46.7; 20.0)	0.42
Anti-depressive treatment			
Yes	137 ± 79		
No	163 ± 72	26.6 (−19.8; 73.1)	0.26
IHD			
Yes	173 ± 76		
No	162 ± 72	−11.1 (−42.7; 20.4)	0.49
Previous stroke			
Yes	175 ± 89		
No	161 ± 71	−14.3 (−44.9; 16.2)	0.36
		*Beta* (95% CI)	*p*-value
Age		0.12 (−0.09; 1.46)	0.08
Hgb		−0.32 (−36.2; −14.4)	<**0.001**
Platelet count		−0.19 (−0.38; −0.07)	**0.004**
Hematocrit		−0.23 (−6.8; −1.9)	<**0.001**
Interval between ictus and blood sample collection (h)		−0.04 (−1.07; 0.54)	0.52
Interval between clopidogrel administration (300-mg bolus) and blood sample collection (h)		−0.07 (−0.06; 0.02)	0.28
Interval between blood sample collection and PRU analysis (min)		−0.02 (−0.16; 0.21)	0.79

*PPI, proton pump inhibitor; IHD, ischaemic heart disease; Hgb, haemoglobin; SD, standard deviation. Significant p-values (<0.05) are highlighted in bold*.

**Figure 2:**
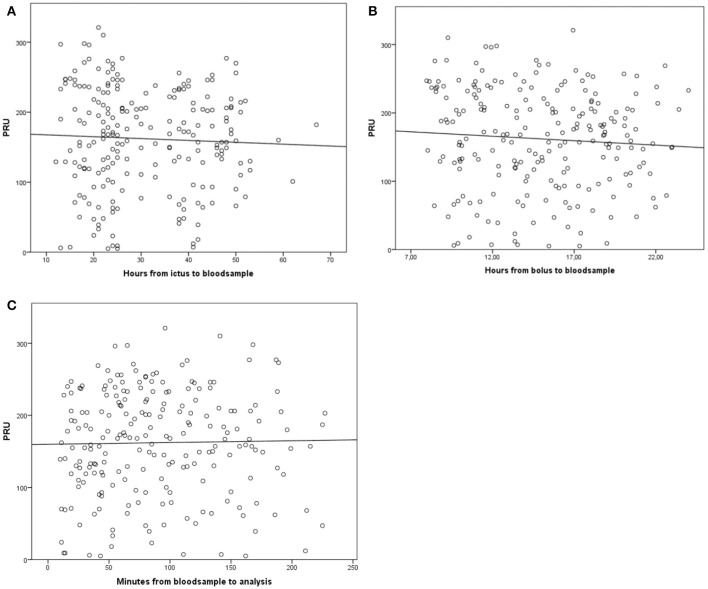
Platelet reaction unit timing results. **(A)** Association of the platelet reaction unit (PRU) value with the interval between stroke onset (ictus) and blood sample collection (h). **(B)** Association of the PRU value with the interval between clopidogrel administration (300-mg bolus) and blood sample collection (h). **(C)** Association of the PRU value with the interval between blood sample collection and PRU analysis (min).

The intervals between ictus and blood collection (*p* = 0.52), clopidogrel bolus administration and blood collection (*p* = 0.28), and blood collection and analysis (*p* = 0.79) did not affect the PRU value (Figure 2).

**Figure 3:**
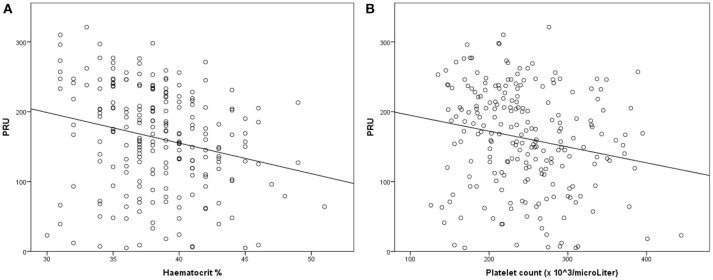
Linear regression analysis for the association of platelet reactivity [measured using Platelet Reaction Units (PRU)] with the haematocrit (Hct) value **(A)** and platelet count **(B)**. Decreased platelet reactivity is associated with an increased Hct value and platelet count.

However, the PRU value was negatively correlated with the platelet count, Hgb level, and Hct value (Figure 3).

In multiple regression analysis, none of the variables except the platelet count (*p* = 0.006) and Hct value (*p* = 0.006) was significant after adjustment for age, sex, the smoking status, anti-hypertensive treatment, PPI treatment, previous stroke history, the presence of IHD, statin treatment, and the presence of diabetes mellitus.

## Discussion

In the present study, 28.8% patients with acute IS or TIA exhibited HTPR after the supervised intake of clopidogrel 300 mg (ensured compliance) in the acute phase after stroke.

According to multivariate analysis, only the Hct value and platelet count influenced HTPR and the PRU value; a decreased Hct value and platelet count resulted in a high PRU value. The VerifyNow system is sensitive to the Hct value (10) and platelet count; therefore, the above finding was probably an indicator of technical issues with the point-of-care device rather than a sign of clopidogrel-induced HTPR.

Our findings are in line with those of a recent meta-analysis, (7) which found that the prevalence of HTPR in clopidogrel-treated stroke patients was 27%. An impaired response to anti-platelet treatment may play a role in the risk of early stroke recurrence and could explain the reason why dual anti-platelet therapies with different modes of action lower the risk of serious vascular events when administered in the acute phase after stroke (11). If previous large randomized controlled trials on the effects of clopidogrel (1, 12) had adjusted for patients with HTPR, the prophylactic efficacy of clopidogrel for patients without HTPR could have been better than anticipated.

### Group differences

In the present study, patients with HTPR after clopidogrel intake were older than those without HTPR (72.8 vs. 67.1 years; *p* = 0.002). This is consistent with previous findings (13) showing diminished anti-platelet effects with increasing age. This could be attributed to the decrease in the activity of several CYP enzymes, including CYP2C19 and CYP1A2, which are responsible for the conversion of clopidogrel to its active metabolite via two-step oxidation, with an increase in age (>65 years) (14).

We also confirmed the previously reported “smokers paradox” in the present study, where the PRU value was lower for smokers than for non-smokers. Some CYP enzymes involved in the oxidation required to convert clopidogrel to its active form (15) are induced by smoking (16). Despite the “smoking paradox”, defined as the effect of smoking on clopidogrel conversion, smoking remains a strong independent risk factor for cardiovascular death (17).

In the present study, the number of patients with HTPR and a high PRU value was higher among patients with diabetes mellitus than among those without. This finding is consistent with the findings of previous studies (18) and is probably a consequence of platelet hyper-reactivity due to platelet anomalies in patients with diabetes (19).

Among the concomitant medications tested in the present study, statins and PPIs appeared to influence HTPR according to univariate analysis, although their effect became insignificant in multivariate analysis. Moreover, these medications did not affect the PRU value. Drugs metabolized by the CYP2C19 pathway, such as omeprazole, esomeprazole, and citalopram, have been suspected of masking the effects of clopidogrel because of CYP2C19 inhibition. Some studies have suggested the use of other PPIs (20). We only evaluated the effects of different PPIs as a single group in our study. If each generic PPI is individually tested, the results could be different. This, however, requires a much larger study.

A recent meta-analysis by Bykov et al. (21) found that a small decrease in the effectiveness of clopidogrel was associated with concomitant exposure to CYP2C19-inhibiting SSRIs. However, none of the patients in our study received CYP2C19-inhibiting SSRIs.

### The verifynow system

The VerifyNow system was chosen as the test device in the present study, because the results obtained with this point-of-care device have previously been correlated with clinical outcomes (22) most recently in a systematic review and meta-analysis of patients with cardiovascular disease (23). Furthermore, this system can evaluate the clopidogrel response in patients receiving dual anti-platelet treatment with clopidogrel and ASA (24).

Several studies have indicated poor treatment compliance as the most important reason for unsatisfactory platelet inhibition (25). In the present study, compliance was ensured because the drug intake by all patients was carefully supervised and documented in the hospital setting.

Accordingly, the poor clopidogrel response could be attributed to biochemical interactions, pharmacokinetics, or, in some cases, genetic factors.

### Limitations

This study has some limitations. The cross-sectional design leaves no opportunity for repeated measurements of the PRU value and provides no information on the clinical outcomes of patients. Both these factors would be very useful in a clinical setting. In addition, sub-group analysis was limited because of the small sample size, and this could have resulted in undetected effects of different variables.

The PPI-subgroup reflects only patients previous treated with PPI at not those prescribed PPIs at time of clopidogrel loading dose. If both premorbid PPI-treated patients and those prescribed PPI at the time of bolus were tested as a group, we might have seen a difference.

Unfortunately, we did not have data on patients with premorbid aspirin use. Even though the VerifyNow is designed to distinguish platelet inhibition with clopidogrel from that of aspirin, this subgroup would still have been interesting because one must assume that these patients have a more premorbid cardiovascular disease. A more standardized approach for the timing of blood collection after clopidogrel intake and an optimal time interval between blood collection and analysis would minimize any concerns about the effects of these time gaps on the study results. However, this study was conducted in an actual clinical setting, and standardization of these time intervals was not possible. Nevertheless, these time intervals did not influence HTPR or the PRU value.

Baseline measurement of the PRU value before the administration of clopidogrel could predict the PRU value after clopidogrel intake. This, however, was not possible in our study, because it would have delayed the time to anti-platelet treatment initiation. Evaluation of other platelet parameters such as immature platelets and thromboxane would also be interesting, although such evaluations are not part of the routine set-up and were consequently omitted in our study. Finally, this study aimed to evaluate the prevalence of non-responders in the immediate acute phase after stroke. We acknowledge that the “long-term” responder status may change; however, this was not within the scope of our study.

In conclusion, our findings showed that the administration of clopidogrel 300 mg in the acute phase after IS or TIA resulted in HTPR in approximately one-third of the patients. The recognition of HTPR to specific anti-platelet agents in the acute phase after stroke may be the first step toward interventions that may further minimize the early recurrent stroke risk. Further large randomized trials including clinical outcome assessments are necessary.

## Author contributions

CR and TW: study conception and design; CR: acquisition of data; CR, TW, and NR-J: analysis and interpretation of data; CR: drafting of manuscript; TW and NR-J: critical revision.

### Conflict of interest statement

The authors declare that the research was conducted in the absence of any commercial or financial relationships that could be construed as a potential conflict of interest.
